# Host outdoor exposure variability affects the transmission and spread of Zika virus: Insights for epidemic control

**DOI:** 10.1371/journal.pntd.0005851

**Published:** 2017-09-14

**Authors:** Marco Ajelli, Imelda K. Moise, Tricia Caroline S. G. Hutchings, Scott C. Brown, Naresh Kumar, Neil F. Johnson, John C. Beier

**Affiliations:** 1 Laboratory for the Modeling of Biological and Socio-technical Systems, Network Science Institute, Northeastern University, Boston, MA, United States of America; 2 Center for Information and Communication Technology, Bruno Kessler Foundation, Trento, Italy; 3 Department of Geography and Regional Studies, College of Arts and Sciences, University of Miami, Coral Gables, FL, United States of America; 4 Department of Public Health Sciences, Miller School of Medicine, University of Miami, Miami, FL, United States of America; 5 School of Architecture, University of Miami, Coral Gables, FL, United States of America; 6 Department of Physics, College of Arts and Sciences, University of Miami, Coral Gables, FL, United States of America; Institute for Disease Modeling, UNITED STATES

## Abstract

**Background:**

Zika virus transmission dynamics in urban environments follow a complex spatiotemporal pattern that appears unpredictable and barely related to high mosquito density areas. In this context, human activity patterns likely have a major role in Zika transmission dynamics. This paper examines the effect of host variability in the amount of time spent outdoors on Zika epidemiology in an urban environment.

**Methodology/Principal findings:**

First, we performed a survey on time spent outdoors by residents of Miami-Dade County, Florida. Second, we analyzed both the survey and previously published national data on outdoors time in the U.S. to provide estimates of the distribution of the time spent outdoors. Third, we performed a computational modeling evaluation of Zika transmission dynamics, based on the time spent outdoors by each person. Our analysis reveals a strong heterogeneity of the host population in terms of time spent outdoors–data are well captured by skewed gamma distributions. Our model-based evaluation shows that in a heterogeneous population, Zika would cause a lower number of infections than in a more homogenous host population (up to 4-fold differences), but, at the same time, the epidemic would spread much faster. We estimated that in highly heterogeneous host populations the timing of the implementation of vector control measures is the major factor for limiting the number of Zika infections.

**Conclusions/Significance:**

Our findings highlight the need of considering host variability in exposure time for managing mosquito-borne infections and call for the revision of the triggers for vector control strategies, which should integrate mosquito density data and human outdoor activity patterns in specific areas.

## Author summary

Zika spread in urban environments is characterized by complicated spatio-temporal patterns, likely linked not only to mosquito density but also to human activity patterns. We conducted a survey on Miami-Dade County residents on time spent outdoors. We then analyzed the Miami-Dade survey data and previously published activity data on the entire U.S. population. Our findings indicate high variability in the amount of time spent outdoors by individual residents. Specifically, we found that the majority of persons spend little time outdoors whereas a few people spend a large amount of time outdoors. This analysis was further elaborated in a modeling framework, showing the effect of the host variability in outdoor time on relevant epidemiological indicators (such as the total number of cases and epidemic timing of spread). Our investigation reveals that when host variability in time spent outdoors is high, Zika will infect fewer people. However, it will spread at a quicker pace than in homogenous host populations (where all people spend nearly the same amount of time outdoors). This information is instrumental for defining new triggers for vector control measures based on both mosquito vector density data and human activity patterns in different areas.

## Introduction

Since Brazil reported its first Zika cases in mid-2015, the epidemic has ramped up to a global scale [1]. This is due to the suitable environmental conditions of tropical and sub-tropical regions around the globe for the highly adaptable arbovirus vectors *Aedes aegypti* and *Aedes albopictus* [2,3] and the large volume of international passengers [4–6]. Different from the long history of major epidemics of mosquito-borne diseases in rural areas and/or in the developing world [7–9], large outbreaks in western countries are often prevented by socioeconomic factors such as lifestyle, housing infrastructure, and sanitation [10]. Nonetheless, these outbreaks do occur in the west. In such a context, identifying specific locations at risk for mosquito-borne infection transmission in urban environments, where transmission events are rare and seemingly unpredictable, poses new challenges for mosquito control and management programs, epidemiologists, and modelers.

A clear example is represented by the transmission of the emerging Zika virus in the United States. In fact, in sharp contrast to what happens in less developed countries where a considerable fraction of the infection occurs inside the home and in its immediate proximity [11,12], Zika dynamics were likely linked to outdoor exposure of hosts to *Aedes* mosquitoes (e.g., for working or leisure activities) [13]. To complicate the picture, it should also be considered that the time spent outdoors is highly subjective [14,15], depending on individual habits, type of employment (whether indoors or outdoors), and times of the day spent at work. For example, it is easy to imagine that there exists a large difference in the amount of time spent outdoors between a construction worker who spends her/his shifts outdoors, and a bank employee.

The purpose of this analysis is to investigate the effect of host variability in outdoor time exposure on the epidemic spread. To fulfill this goal, we: i) performed a survey on time spent outdoors in Miami-Dade County, Florida residents; ii) analyzed both the obtained survey results and previously published data on the U.S. population [14] to quantify the heterogeneity level; and iii) developed a simple dynamical model of the transmission process of a vector-borne disease, explicitly accounting for host variability in the time spent outdoors. The model is calibrated on the basis of the most recent knowledge on the Zika infection timeline [16] and transmissibility [16–19]. The performed modeling analysis quantifies the effect of host variability in time spent outdoors on epidemiological indicators such as the final epidemic size (i.e., the fraction of the population that becomes infected over the whole course of an epidemic), epidemic timing, and outbreak probability. The 2016 Zika epidemic in the U.S. showed a complicated spatiotemporal pattern, characterized by multiple waves of infections, long tails of the distribution, and dramatic variability in the number of reported human cases at the neighborhood level [20]. That pattern was surely partially due to the complicated ecology of the vector *Ae*. *aegypti* (e.g., short flight-range, need of specific breeding sites, outdoor resting habitats, and sugar-feeding sources) [21–24]. However, we believe that human behaviors such as mobility at the micro scale (e.g., to seek places for leisure activities) and time spent outdoors exposed to mosquito bites have contributed to the observed complex pattern. Explicitly modeling host variability in exposure time might help shed light on the observed highly heterogeneous pattern of spread and might be useful in informing mosquito control strategies for controlling Zika epidemics in a complex urban environment to maximize efficiency.

## Methods

### Ethics statement

The survey was approved on June 7, 2016 by the University of Miami Institutional Review Boards (IRB ID: 20160466). Only individuals of at least 18 years of age were sampled. All subjects provided oral informed consent.

### Data collection

A structured bilingual (English and Spanish) telephone questionnaire was designed and administered by trained graduate students. The first part of the questionnaire included knowledge of Zika virus and severity of disease. The second part had questions on preventive measures and practices including questions that asked “on an average weekday, about how many hours per day do you spend outside? and “on an average weekend, about how many hours per day do you spend outside?” The questionnaire was administered to 282 randomly selected Miami-Dade heads of household or other household members of at least 18 years of age in the absence of the household head using random Digit Dialing (RDD). The surveys took 10–15 minutes to complete. The interviews were conducted in July through December 2016 through Qualtrics software. Administration of questionnaire was monitored daily for quality control of all trained graduate students’ work. Specifically, following each monitored interview, the second author (I.K.M.) met privately with the monitored student interviewer to discuss aspects of the interview and to point out any verbal errors or procedural errors for the student to improve upon, and most interviewers improved after ongoing feedback. Phone numbers of Miami residents were purchased from Survey Sampling International. Descriptive statistics of study participants are reported in S1 Table.

### Data analysis

Together with our survey data for Miami-Dade County, Florida, we also analyzed previously published data on time spent outdoors by the U.S. population [14]. A gamma distribution was fitted to each of the available datasets. We used a gamma distribution as it is a suitable broad tailed distribution, which can arise from multiplicative type models and scheduling; similarly broad distributions have been observed in human activity times in other areas of life [25], which lends support to this particular choice. However, the use of other flexible distributions would lead to similar general conclusions. The posterior distributions of the parameters of the gamma distributions were determined by exploring with a MCMC (Metropolis–Hastings) algorithm [26] the likelihood of candidate parameters.

### The model

**Fig 1:**
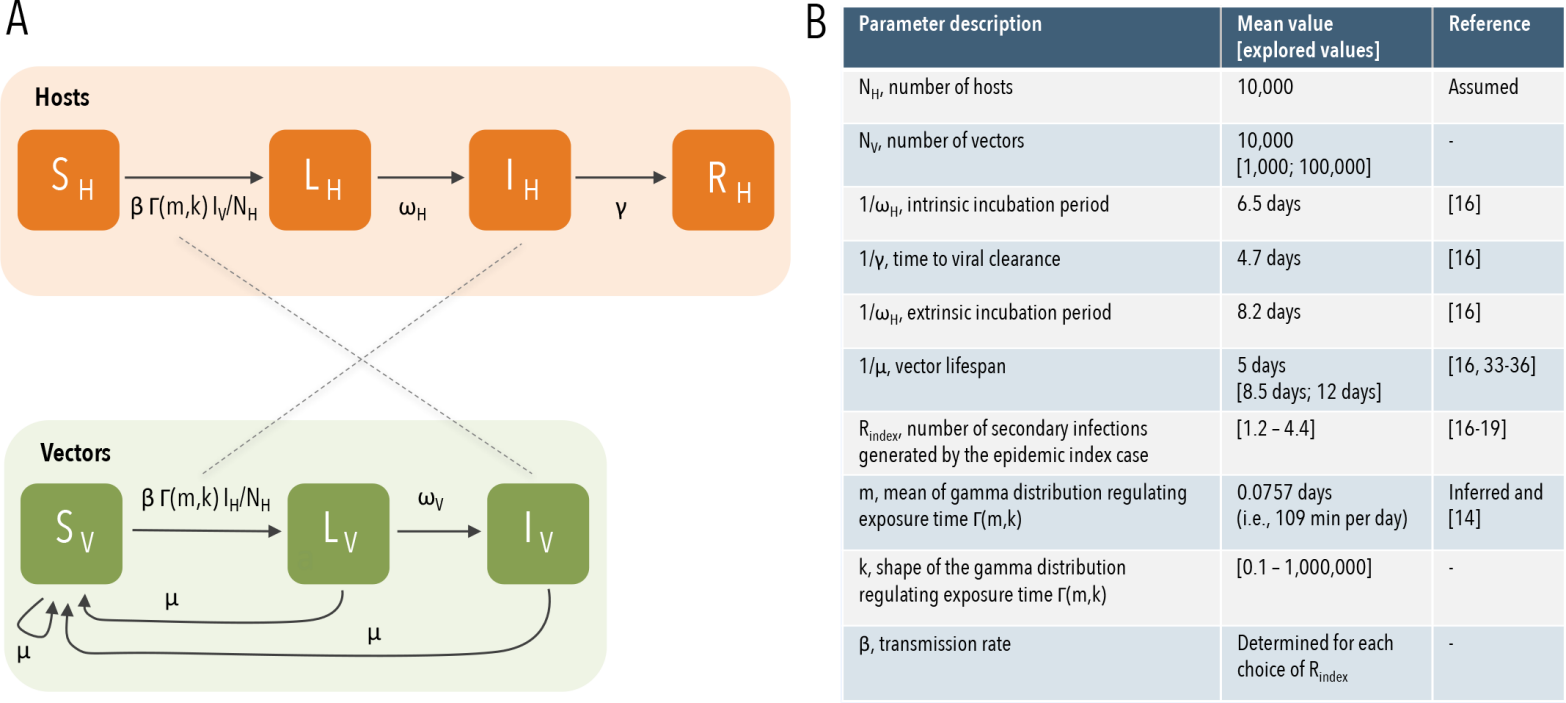
Epidemic flow and model parameters. **A** Schematic representation of the infection transmission process. S_H_, L_H_, I_H_, and R_H_ represent susceptible, latent, infectious, and recovered human hosts, respectively; S_V_, L_V_, and I_V_ represent susceptible, latent, and infectious mosquitoes, respectively; parameters are described in panel B. **B** Description of model parameters and values used in the simulations.

We developed an individual-based dynamical model of Zika transmission. Each human host is explicitly considered in the model and has associated: i) an epidemiological status (namely, susceptible, latent, infectious, and recovered); and ii) the amount of time that she/he is exposed per day to mosquito bites, corresponding to the amount of time spent outdoors (referred in the following as “exposure duration”). Each mosquito vector has an epidemiological status, namely, susceptible, latent, and infectious. As we are interested only in assessing the effect of the variability in the human population of exposure durations, we deliberately decided to keep the model as simple as possible. For the ease of simplicity, in the absence of interventions, we keep the mosquito population constant over time and omit the complex life cycle of mosquitoes, which depends on environmental factors such as temperature, presence of breeding sites, outdoor resting habitats, and sugar-feeding sources [23,24]. Although this is a strong assumption, it will help us in isolating the effect of the variability of the duration of the host exposure from the possible confounding effects of mosquito population dynamics. A schematic representation of the model is presented in Fig 1A.

Keeping in mind the above assumption, we describe the transmission model as follows. At each time step (representing 1 day) of the simulation *t*, a susceptible host *i* has a probability $1-exp\{-\sum_{j=1}^{N_{V}}\Gamma_{i}(m,k)\beta \frac{I_{V}^{j}(t)}{N_{V}}\frac{N_{V}}{N_{H}}\}$ of becoming latent, where:

- $I_{V}^{j}(t)$ is 1 if vector *j* is infectious at time *t*, 0 otherwise;
- N_V_ is the total number of vectors in the population;
- N_H_ is the total number of human hosts in the population;
- β is the transmission rate;
- Γ_i_(m,k) is the duration of the exposure of host *i*, which is sampled (one time per simulation for each person) from a gamma distribution of mean *m* and shape parameter *k*.

Intuitively, the probability of getting the infection is 1 minus the product of avoiding the infection from bites of each mosquito vector. Note that the model assumes a constant biting rate during the exposure time. The ratio N_v_/N_H_ is a consequence of the fact that female mosquitoes only take a fixed number of blood meals per unit of time. As such, the net transmission rate is held to an upper limit, which does not depend on the absolute abundance of mosquitoes and hosts–that is, the biting rate times the number of female mosquitoes per person [27,28].

As in the classic theory of infectious disease modeling [27], we make the simplest possible assumption and model the amount of time a human host spends in the latent and infectious status by sampling them from two exponential distributions having as mean the duration of the intrinsic incubation period and the time to clearance, respectively.

As regards the infection progress in vectors, at each time step of the simulation, a susceptible vector *i* has a probability $1-exp\{-\sum_{j=1}^{N_{H}}\beta \frac{\Gamma_{j}(m,k)I_{H}^{j}(t)}{N_{H}}\}$ of becoming latent, where $I_{H}^{j}(t)$ is 1 if host *j* is infectious at time *t*, 0 otherwise. The amount of time a mosquito spends in the latent status is sampled from an exponential distribution having the duration of the extrinsic incubation period as mean. Irrespective of the epidemiological status, the lifetime of vectors is sampled from an exponential distribution having vector lifetime as mean. Finally, at each time step of the simulation *t*, new mosquitoes emerge as susceptible adults at the same rate as they die, μ, which corresponds to the inverse of the duration of mosquito lifespan.

Essentially, this model accounts for two important features: i) hosts can transmit and acquire the infection from mosquitoes only when they are exposed to mosquito bites; and ii) the exposure duration for the hosts might be variable in the population, i.e., it strongly depends on the individual characteristics and behaviors of a host.

We measured the transmission potential of the infection in terms of R_index_, which is defined for a vector-borne disease as the average number of secondary hosts infected by the first infected host through the means of infected vectors. As shown in the literature [29–32], R_index_ represents a good proxy for the basic reproduction number and, at the same time, it is easier to define for individual-based models.

Model simulations are initialized by introducing one infectious host in a fully susceptible population of both hosts and mosquitoes. Given the stochastic nature of the model, results are based on 10,000 realizations. The model is calibrated on the basis of the recent findings on Zika timeline of infection of Ferguson and colleagues [16], Zika reproduction number [16–19], and *Ae*. *aegypti* lifespan [16,33–36]. The values of model parameters used in the simulations are reported in Fig 1B where, as baseline scenario we assume a 1:1 ratio between hosts and vectors and 5 days as mosquito lifespan. It is important to note that even across an area as small as a city, there are likely differences both in human activities, with areas characterized by larger probabilities of outdoor activities (for instance because of the presence of parks, beaches, athletic fields) and in mosquito life history traits (e.g., mosquito densities, biting rates and lifespan). However, properly characterizing these differences is a hard task that would require the collection of highly specific environmental, socio-economic, and entomological data. In order to explore the effects of such variability, we performed an in-depth sensitivity analysis on the shape parameter of the distribution regulating host exposure time to mosquito bites, R_index_ (which entails differences in the biting rate), the ratio between hosts and vectors, and mosquito lifespan.

We simulate the effect of simple interventions aimed at reducing adult mosquitoes abundance. Specifically, we assume an instantaneous reduction of 80%, 90%, or 99% of the mosquito adult population (irrespective to the infection state) at the day of intervention–namely, 0, 30, 60, or 90 days after the introduction of the first infectious person. The explored range for the magnitude of the vector control strategies is in line with empirical studies estimating a 91% decrease in the density of *Ae*. *aegypti* after the deployment of adult traps [37].

## Results

### Respondent characteristics

Of the 400 phone interviews administered, 282 responded and 270 were completed and used in the study, a response rate of 71%. Fifty-five percent of surveyed respondents were female and 41% were male. Their ages ranged from 18 to 94, with a mean age of 48.9 and a standard deviation of 18. Over half (52.5%) of the respondents were foreign born, 61% considered themselves Hispanic, and 53% were married. Forty-seven percent held fulltime employment. Sixteen percent of respondents in this survey reported household incomes less than $25,000 and 18% reported incomes greater than $100,000. More than one-quarter of respondents (27.7%) reported having a Bachelor’s degree and 19.1% had a General Education Diploma or GED. At the time of the interview, less than half of the respondents (42.6%) reported spending between 2 and 5 hours outside on an average weekday while almost half of the respondents (47.9%) spent between 2 and 5 hours outside on an average weekend. Summary statistics of the study participants are reported in S1 Table.

### Time spent outdoors

**Fig 2:**
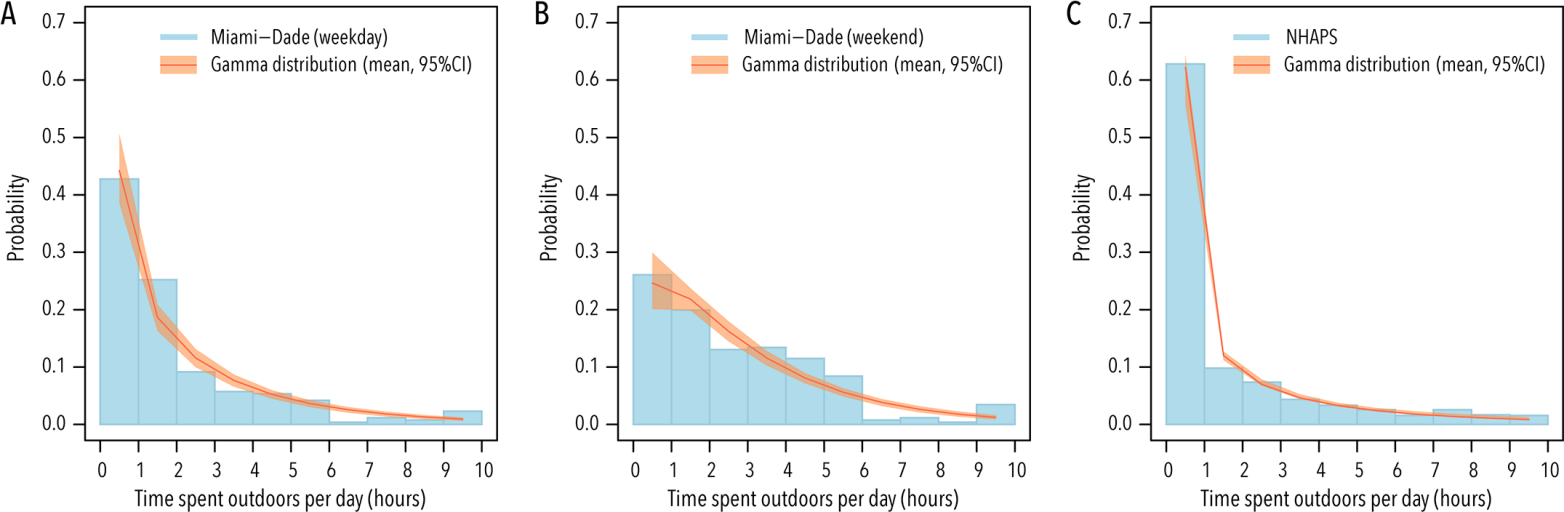
Amount of time spent outdoors. **A** Amount of time spent outdoors per day in weekdays in the surveyed Miami-Dade County population and the posterior gamma distribution. For the sake of clarity, the plot shows only outdoors time up to 10 hours; however, the computation of the likelihood took into account all observations (including those where the outdoors time is larger than 10 hours). **B** As A, but for weekends. **C** As A, but data are taken from the NHPS survey on the U.S. population [14].

We analyzed the self-reported time spent outdoors in Miami-Dade county resident population during weekdays and during weekends. The collected dataset used for this analysis is available in S2 Table. The posterior distribution of mean and shape of the gamma distribution regulating the amount of time spent outdoors are *m* = 135 minutes per day (95%CI: 116–158) and shape parameter *k* = 0.66 (95%CI: 0.52–0.81) for weekdays (Fig 2A). The figure becomes *m* = 177 minutes per day (95%CI: 158–198) and shape parameter *k* = 1.21 (95%CI: 0.98–1.46) for weekends (Fig 2B). This highlights a remarkable difference on the average time spent outdoors during weekdays and weekends (43 min more on weekends) and weekdays have a more skewed distribution.

Interestingly, we found similar results to those obtained for Miami-Dade County residents for weekdays when we analyzed the National Human Activity Patterns (NHAPS) survey data on the U.S. population [14]. Specifically, from the U.S. national sample we estimated *m* = 109 minutes per day (95% CI: 100–157) and shape parameter *k* = 0.31 (95% CI: 0.29–0.36) (Fig 2C).

These results suggest high level of host variability in the time spent outdoors, which can be modeled by skewed gamma distributions with shape parameters lower than 1.5. Next we will present results of the entire spectrum of variability in terms of time spent outdoors. In other words, we did not fix *k* to the values we have estimated. Rather, our model-based evaluation considers *k* in the range 0.1-∞ (and keeps fixed *m* = 109 minutes, as variations in the mean exposure duration embedded in different values of R_index_). Note that *k* = ∞ corresponds to a situation where all people spend the same amount of time *m* outdoors, and low values of *k* correspond to skewed distributions (i.e., the majority of persons spend little time outdoors and few persons spend a large amount of time outdoors).

### Variability in the duration of host outdoor exposure and Zika spread

**Fig 3:**
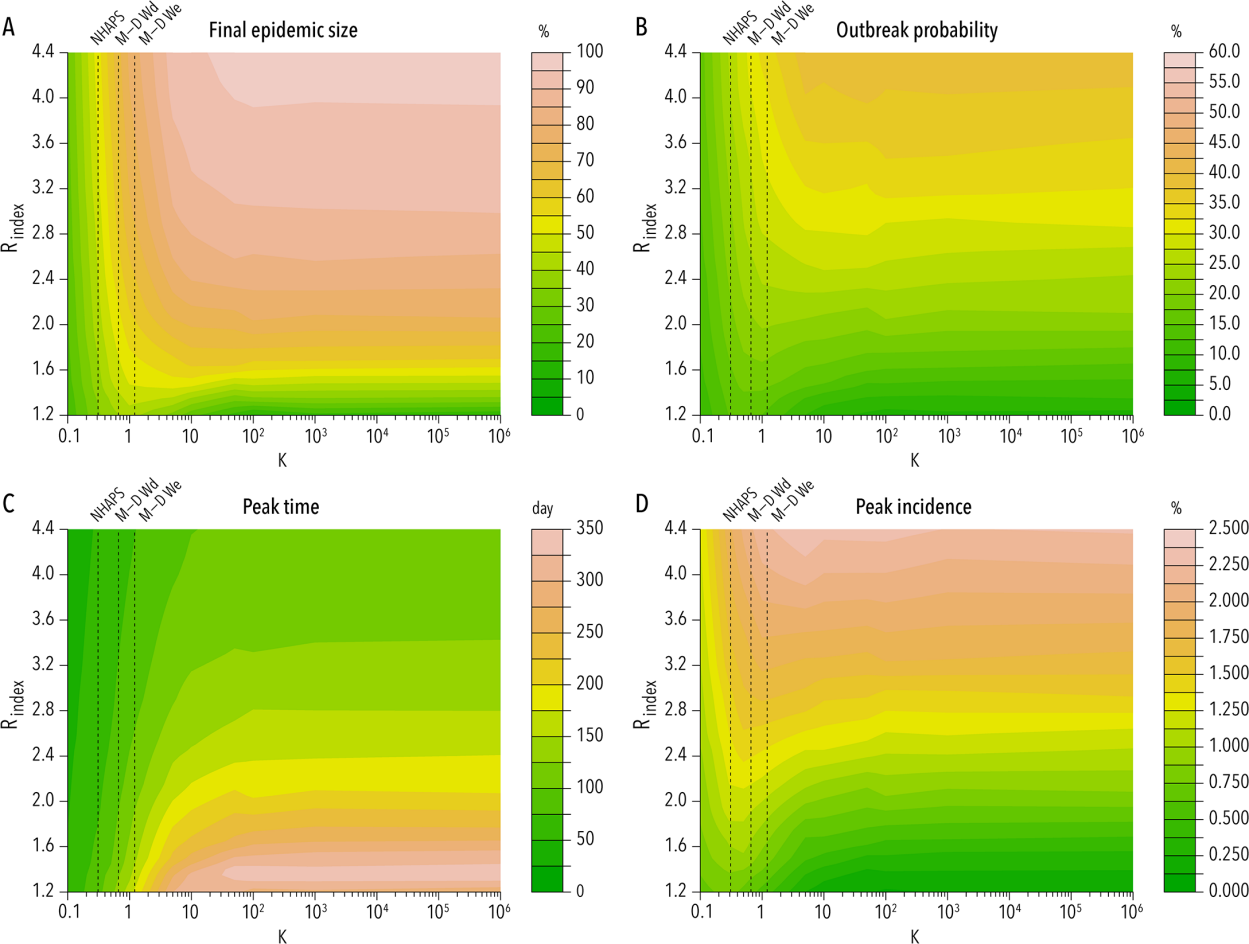
Effect of variability in the duration of host outdoor exposure on basic epidemiological indicators. **A** Estimated mean final epidemic size as a function of k and R_index_. Vertical dashed line corresponds to the mean value of k obtained by analyzing NHAPS data [14] (NHAPS), Miami-Dade County residents data for weekdays (M-D Wd), and Miami-Dade County residents data for weekends (M-D We). Model parameters are assumed at their baseline value as reported in Fig 1B, and N_V_ = 10,000. **B** As A, but for the outbreak probability. We define “outbreak” an epidemic of at least 100 cases. **C** As A, but for the peak time. **D** As A, but for the peak day incidence.

The parameter determining the dispersion of the distribution regulating the exposure duration (*k*) has a dramatic effect on the final epidemic size, especially for R_index_>1.6 (Fig 3A). For instance, for R_index_ = 3 the estimated final epidemic size is 27.2%, 57.1%, and 90.1%, for *k* = 0.1, *k* = 0.5, and *k* = ∞, respectively, suggesting that the higher is the variability in exposure duration (in a given population), the lower is the epidemic size. This can be interpreted by considering that high heterogeneity means that a large fraction of the population spends very little time outdoors and is thus exposed to a small risk of infection. What is of interest here is that the values of the shape parameter best fitting survey data are in the range 0.29–1.68, supporting our hypothesis of a highly variable distribution of the duration of the exposure. In particular, our estimates lie inside a region of the parameter space where the estimated final epidemic size is remarkably different from what is obtained by assuming a homogenous population as in the classic modeling theory (Fig 3A). Such a pattern is not obvious *a priori*. In fact, from one side, a highly variable duration of the exposure implies that only a fraction of the population has a large risk of infection; from the other side, a highly variable exposure duration implies that there is a lower number of human hosts exposed to mosquito bites and thus a higher probability for mosquitoes to blood-feed on infected hosts and become infected. In fact, interestingly, for low values of R_index_ (approximately lower than 1.4) we observe that the final epidemic size can be larger in heterogeneous populations (0.2<k<1) than in fully homogenous or extremely heterogeneous populations (Fig 3A).

Variability in host exposure duration has remarkable consequences for the outbreak probability, which is generally lower in a highly heterogeneous population, although for low values of R_index_ the situation could be different (Fig 3B). This implies that in situations suggested by the human activity data, epidemics are less likely to start. However, our modeling results show that in highly heterogeneous populations once the epidemic is started, it tends to spread much more quickly (Fig 3C) and with a relatively larger peak incidence, especially for low-intermediate values of R_index_ (approximately lower than 3) (Fig 3D). Remarkably, this gives far less time to perform intervention measures such as removal of breeding sites, larval control, and outdoor spraying of adulticides.

**Fig 4:**
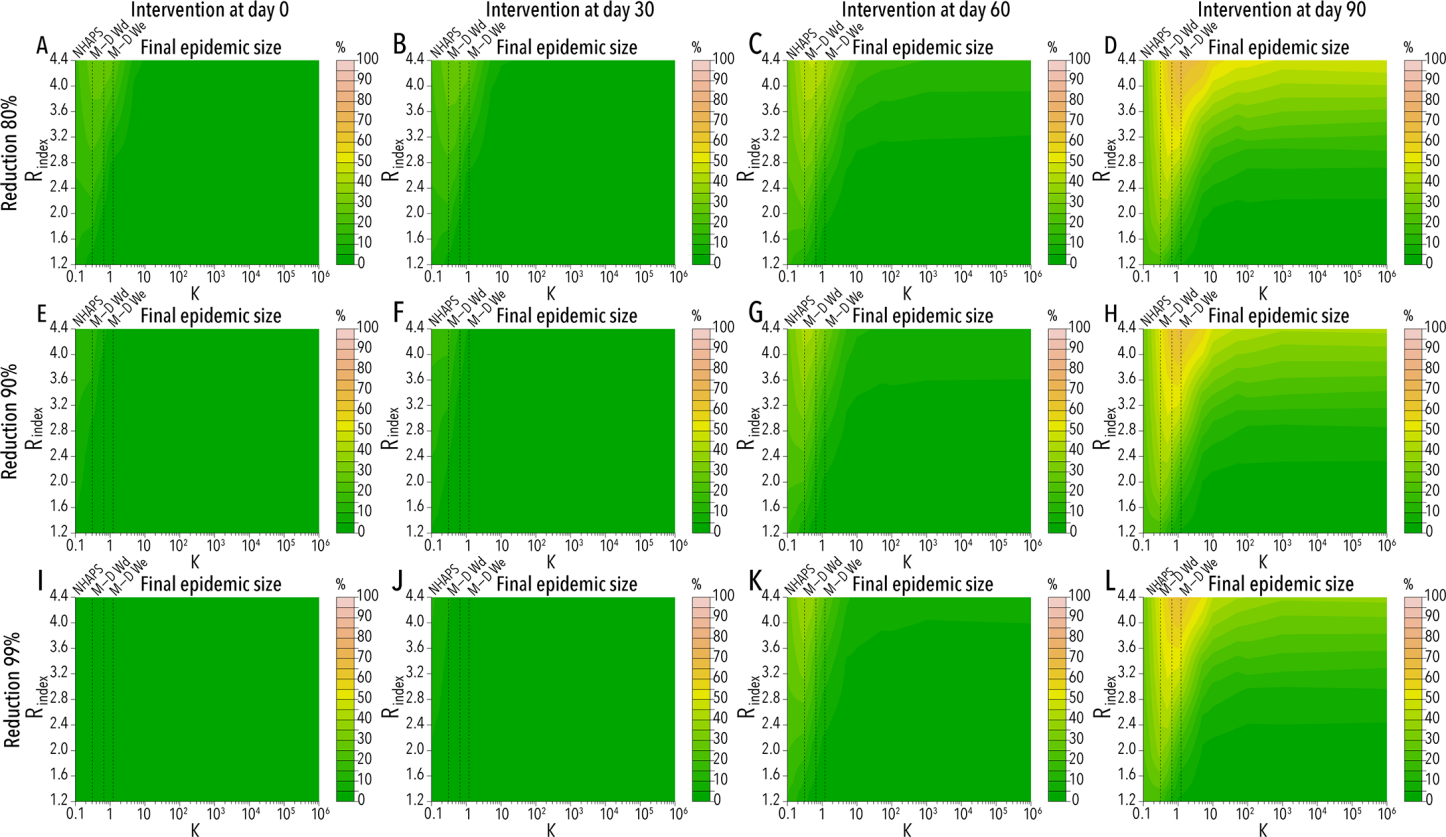
Effectiveness of mosquito control measures. **A** Estimated mean final epidemic size as a function of k and R_index_ by assuming an intervention on day 0 capable of reducing mosquito adult population by 80%. Vertical dashed line corresponds to the mean value of k obtained by analyzing NHAPS data [14] (NHAPS), Miami-Dade County residents data for weekdays (M-D Wd), and Miami-Dade County residents data for weekends (M-D We). Model parameters are assumed at their baseline value as reported in Fig 1B, and N_V_ = 10,000. **B** As A, but the intervention starts on day 30. **C** As A, but the intervention starts on day 60. **D** As A, but the intervention starts on day 90. **E** As A, but the reduction of mosquito population is 90%. **F** As E, but the intervention starts on day 30. **G** As E, but the intervention starts on day 60. **H** As E, but the intervention starts on day 90. **I** As A, but the reduction of mosquito population is 99%. **J** As I, but the intervention starts on day 30. **K** As I, but the intervention starts on day 60. **L** As I, but the intervention starts on day 90.

By considering an intervention leading to an instantaneous reduction of the adult mosquito population, we were able to quantify the reduction of the epidemic final size for different levels of heterogeneity, R_index_, and timing and efficacy of mosquito control interventions (see Fig 4). Our results clearly show that the timing of the intervention is far more important than the achieved reduction of the vector population when the duration of the exposure is highly variable (as is the case of Miami-Dade residents and the U.S. population), especially for high values of the R_index_.

Finally, we performed a sensitivity analysis to evaluate to what extent results are affected by the ratio of hosts and vectors and by mosquito lifespan. The qualitative patterns that we have discussed so far for the baseline scenario (where the ratio of hosts to vectors is 1:1) are confirmed by this sensitivity analysis (S1 Fig). From a quantitative point of view, changing the human to vector ratio has dramatic consequences on the outbreak probability after the introduction of a single infected host. As in the classic modeling theory, larger outbreak probabilities are observed when the human to vector ratio is low [28,38]. Mosquito lifespan has little impact on the final attack rate of the epidemic and on the outbreak probability, while it heavily affects the timing of epidemic spread: the longer the mosquito lifespan, the slower the epidemic spreads (S2 Fig).

## Discussion

We analyzed both survey activity data of the resident population of Miami-Dade County, Florida collected in July through December 2016, and previously published data on the U.S. population to determine whether the variability in the duration of outdoor exposure is an important factor of the infection transmission process. In particular, we are interested in understanding how it might shape the timing and final size of an epidemic–both important topics in the context of determining the effectiveness of intervention control strategies. We found that time spent outdoors is highly variable, with the large majority of the population spending very little time outdoors. We observed that the final epidemic size decreases as the heterogeneity in exposure time increases. This finding suggests that time spent outdoors is an important variable that should be considered in Zika preventive strategies, particularly in areas where *Aedes* mosquitoes have to find hosts outdoors for blood feeding. Notably, our modeling results also show that when the heterogeneity in exposure duration is large, epidemics spread much faster. This implies that public health decision-makers will need to have the capacity to respond quickly and adequately to sudden mosquito threats (e.g., plan and perform mosquito control interventions such as larval control and outdoor spraying of adulticides in a timely manner). These results seem to correspond well to those reported elsewhere, suggesting that broad-tailed inter-event times slow down spreading phenomena [39,40]. Our results are also in line with the well-documented skewed distribution of the number of secondary infections caused by a primary infection (through interactions with the vector) [38,41]. However, previous studies on malaria, dengue, and chikungunya showed that this skewed distribution and the high spatial clustering are linked to human exposure to mosquito bites in the household setting [11,12,42,43]. On the other hand, there is empirical evidence supporting that, in contexts where the transmission mainly occurs outdoors, the distribution of secondary infections and the spatial clustering are linked to human behaviors [44], as hypothesized in our analysis.

The epidemiological investigation on the local Zika outbreak conducted in July 2016 in Miami-Dade County provides evidence of relatively high risk of infection for outdoor workers [13]. Moreover, a recent survey conducted in Miami-Dade County construction industry employees highlighted the vulnerability to Zika infection of some categories of people who spend a considerable time outdoors and are exposed to mosquito bites on a regular basis [45]. Both studies provide further support to the relevance of our analysis.

Another important public health implication of our study is that vector control operations are usually directed to areas with the highest vector densities on the basis of entomological surveillance. Instead, here we highlight how it is the combination of vector densities and biting rates (as well as other entomological characteristics such as mosquito lifespan), and human exposure time outdoors that is key to Zika infection risk. Consequently, operation control efforts could be prioritized and directed toward areas characterized by high levels of human outdoor activities, such as recreational areas and tourist attractions, rather than, for instance, on residential areas. A clear example of such a phenomenon was the 2016 Zika outbreaks in Miami-Dade County, where the initial two affected areas of Wynwood and Miami Beach neighborhoods [46] are also preferred tourist destinations and well-known areas for outdoor events and activities. This highlights the need to derive new indices to be considered by operational mosquito control programs, categorizing neighborhoods on the basis of both mosquito surveillance counts and human outdoor exposure risk.

Our study had limitations. First, our analysis does not have the rigor to provide any specific operational indications on vector control measures. There are still many unanswered questions about key aspects of mosquito dynamics, transovarial transmission, spatial variability, presence of standing water, importation of cases, and mobility of the human population. Further work is required to develop and calibrate a detailed model of the infection transmission, accounting for all aspects that could provide quantitative insights on specific mosquito control options. Second, in the analyzed datasets on outdoor time, we did not have data to report the reasons as to why people spend time outdoors. Additionally, the differing contexts underlying time spent outdoors is not distinguished among individual persons, although such difference may exist. For instance, between a person who works outdoors for 4 hours per day and another person who spends 4 hours outside for leisure. Our modeling investigation did not account for such an aspect of differing environmental contexts across people with the same amount of time outdoors, although it might affect Zika transmission as the biting activity of *Aedes* mosquitoes is not constant over the course of the day [47]. Moreover, it is well known that extreme temperatures affect Ae. mosquitoes biting rate [48] and they might influence human behavior as well, for instance by keeping people indoors or outdoors. This topic surely warrants further investigations as it might be key for a deeper understanding of Zika transmission.

Notwithstanding these limitations, our model can be considered a good reorientation of the infection transmission process in contexts where mosquitoes depend very little (or even not at all) on finding hosts indoors, as they must seek and feed on them outdoors. This is a completely different situation as compared to that observed in less developed countries where clusters of cases are often observed around individual households as mosquito blood feeding mainly occurs indoors or in the immediate proximity of houses [12]. On the other hand, the 2016 Zika epidemic in Miami, Florida represents a clear example where the developed model could help in explaining the observed dynamics and provide useful insight for epidemic control.

In conclusion, our analysis shows the non-trivial role of variability in exposure duration to mosquito bites in the dynamics and control of mosquito borne infections. For instance, in a highly heterogeneous population in terms of exposure time, Zika would cause a lower number of infections than in a more homogenous population. But, at the same time, the epidemic would spread much faster, thus leaving a smaller time window to implement control strategies. Our findings highlight the need to consider host variability in exposure time when dealing with mosquito-borne infections and call for a deeper understanding of time people spend outdoors in terms of duration, time of the day, day of the week and activity/location. Moreover, our results can be instrumental for the definition of new indices triggering vector control activities, accounting for both mosquito density and area-specific human outdoor activity patterns. Our modeling analysis is useful for providing general indications and can be easily applied to other settings and mosquitoes-borne diseases such as dengue, chikungunya and yellow fever. However, it is of particular importance for Zika virus, as more than 80% of infected persons do not show symptoms [49], and thus carry on with their lives and spend time outdoors, enhancing the likelihood of Zika transmission.

## Supporting information

S1 Table Descriptive statistics of the survey sample. Supporting table reporting the characteristics of the sample of Miami-Dade County residents.
(PDF)

S2 Table Dataset on Miami-Dade County residents. Supporting table reporting the full dataset on Miami-Dade County residents.
(XLSX)

S1 Fig Sensitivity analysis on human hosts to vector mosquitoes ratio. **A** Estimated mean final epidemic size as a function of k and R_index_. Vertical dashed line corresponds to the mean value of k obtained by analyzing NHAPS data [14] (NHAPS), Miami-Dade County residents data for weekdays (M-D Wd), and Miami-Dade County residents data for weekends (M-D We). Model parameters are assumed at their baseline value as reported in Fig 1B, and N_V_ = 1,000. **B** As A, but for N_V_ = 100,000. **C** As A, but for the outbreak probability. We define “outbreak” an epidemic of at least 100 cases. **D** As C, but for N_V_ = 100,000.
(TIF)

S2 Fig Sensitivity analysis on mosquito lifespan. **A** Estimated mean final epidemic size as a function of k and R_index_. Vertical dashed line corresponds to the mean value of k obtained by analyzing NHAPS data [14] (NHAPS), Miami-Dade County residents data for weekdays (M-D Wd), and Miami-Dade County residents data for weekends (M-D We). Model parameters are assumed at their baseline value as reported in Fig 1B, but for mean mosquito lifespan that is assumed 8.5 days **B** As A, but assuming mean mosquito lifespan equal to 12 days. **C** As A, but for the outbreak probability. We define “outbreak” an epidemic of at least 100 cases. **D** As C, but assuming mean mosquito lifespan equal to 12 days. **E**, As A, but for the peak time. (Note that the scale is different from that of Fig 3C). **F**, As E, but assuming mean mosquito lifespan equal to 12 days.
(TIF)
