# Supplementary material for: Host outdoor exposure variability affects the transmission and spread of Zika virus: Insights for epidemic control
Source: PLoS Negl Trop Dis. 2017 Sep 14;11(9):e0005851. doi: 10.1371/journal.pntd.0005851 (PMC5598931; doi:10.1371/journal.pntd.0005851)
Supplement: S1 Table — Supporting table reporting the characteristics of the sample of Miami-Dade County residents. (PDF) [file pntd.0005851.s001.pdf]

Table S1. Characteristics of Study Participants

Respondent Characteristic	Frequency	Percent (%)
**Gender**		
Male	115	41
Female	155	55
**Age (Mean age= 48.9; range 18-94)**		
Younger than age 30	50	17.7
30-39	25	8.9
40-49	46	16.3
50-59	57	20.2
60-69	42	14.9
70+ years	31	11
**Marital Status**		
Single	66	23
Married	148	53
Separated	5	2
Divorced	25	9
Windowed	16	6
**Do you consider yourself Hispanic?**		
Yes	172	61
No	89	31.6
**Yearly Income (2015)**		
Less than $25, 000	44	16.0
$25,000-$50,000	54	19.1
$50,000-$75,000	38	13.5
$75,000-$100,000	28	9.9
Over $100,000	52	18
**Employment Status**		
Fulltime	131	47
Part time or “semi-retired”	14	5
Retired	60	21.3
Unemployed	18	6.4
Student	14	5
Student & Full-time/Part-time	7	2.5
**Education**		
Less than high school education	6	2.1
General Education Diploma or GED	54	19.1
Some college, including Associate degree	63	22.3
Bachelor’s degree	78	27.7
Some graduate school	10	3.5
Graduate degree or higher	48	17
**Foreign Born**		
Yes	148	52.5
No	112	39.7
**Average hours per day spent outside on a weekday**		
Less than an hour	113	40.1

2-5 hours	120	42.6
5-10 hours	19	6.7
10 or more hours	11	3.9
**Average hours per day spent outside on a weekend**		
Less than an hour	34	12.1
2-5 hours	135	47.9
5-10 hours	26	9.2
10 or more hours	10	3.5
