# Supplementary figures and images for: Host outdoor exposure variability affects the transmission and spread of Zika virus: Insights for epidemic control
Source: PLoS Negl Trop Dis. 2017 Sep 14;11(9):e0005851. doi: 10.1371/journal.pntd.0005851 (PMC5598931; doi:10.1371/journal.pntd.0005851)

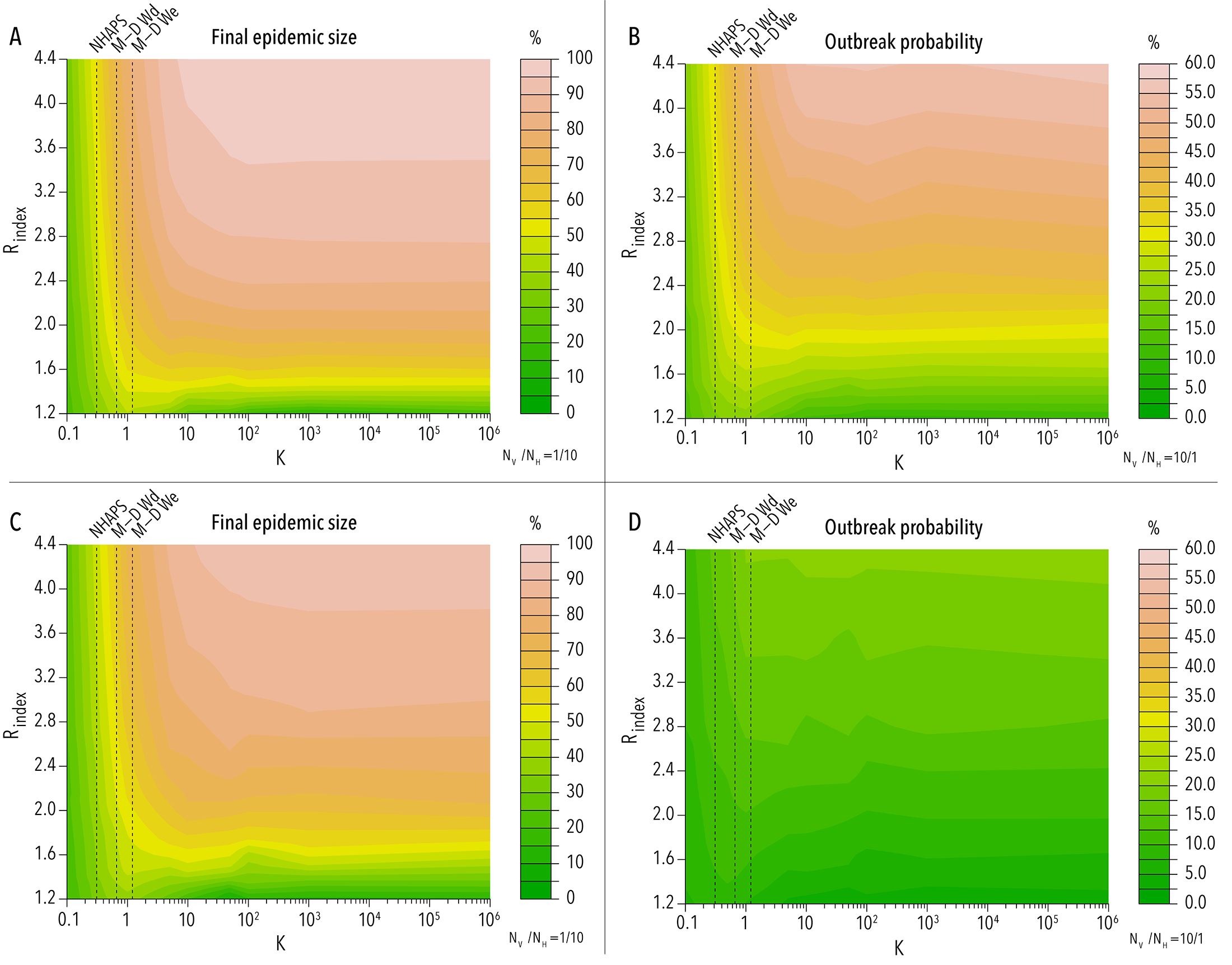

Supplement: S1 Fig — A Estimated mean final epidemic size as a function of k and Rindex. Vertical dashed line corresponds to the mean value of k obtained by analyzing NHAPS data [14] (NHAPS), Miami-Dade County residents data for weekdays (M-D Wd), and Miami-Dade County residents data for weekends (M-D We). Model parameters are assumed at their baseline value as reported in Fig 1B, and NV = 1,000. B As A, but for NV = 100,000. C As A, but for the outbreak probability. We define “outbreak” an epidemic of at least 100 cases. D As C, but for NV = 100,000. (TIF) [file pntd.0005851.s003.tif]

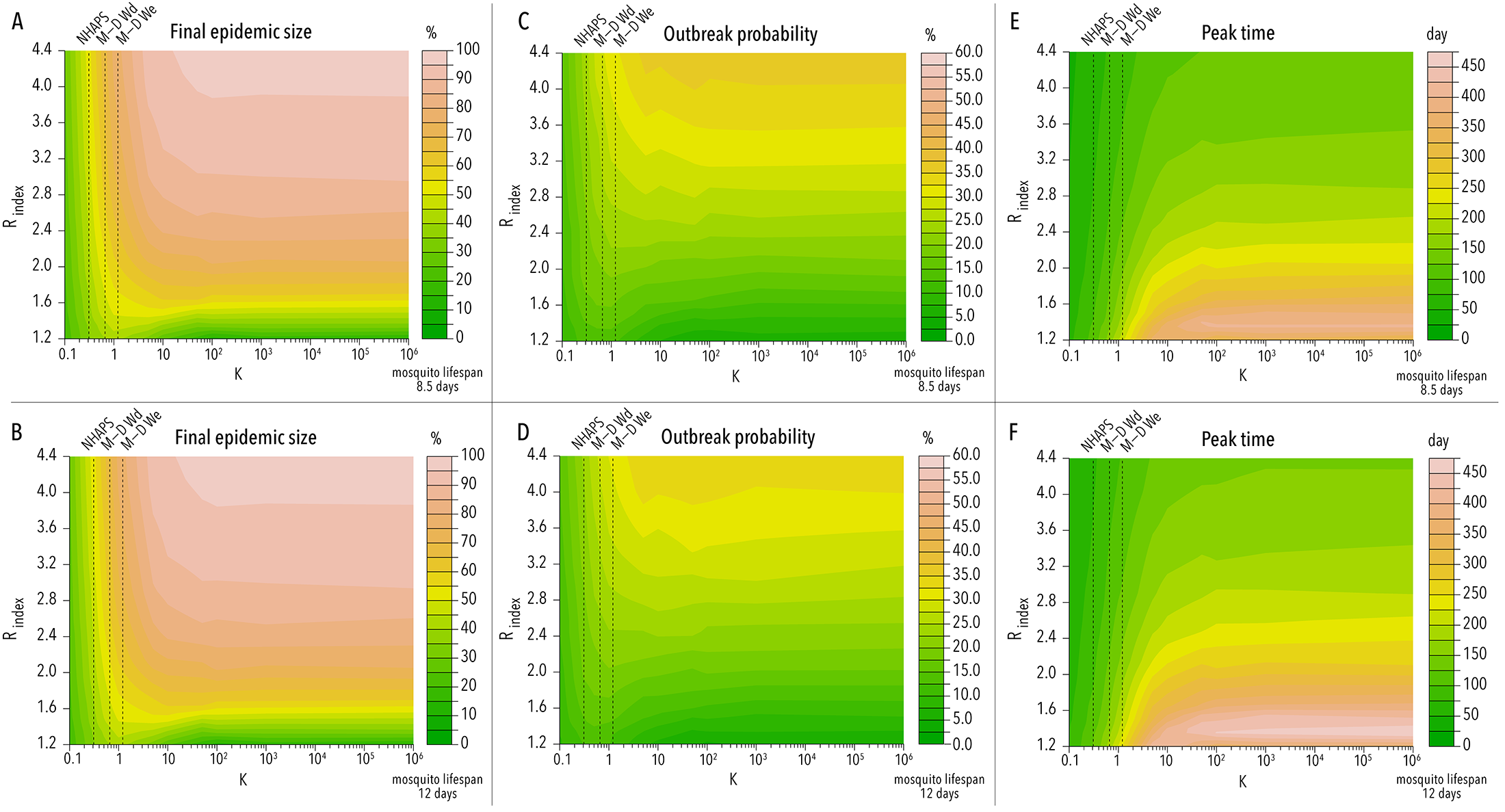

Supplement: S2 Fig — A Estimated mean final epidemic size as a function of k and Rindex. Vertical dashed line corresponds to the mean value of k obtained by analyzing NHAPS data [14] (NHAPS), Miami-Dade County residents data for weekdays (M-D Wd), and Miami-Dade County residents data for weekends (M-D We). Model parameters are assumed at their baseline value as reported in Fig 1B, but for mean mosquito lifespan that is assumed 8.5 days B As A, but assuming mean mosquito lifespan equal to 12 days. C As A, but for the outbreak probability. We define “outbreak” an epidemic of at least 100 cases. D As C, but assuming mean mosquito lifespan equal to 12 days. E, As A, but for the peak time. (Note that the scale is different from that of Fig 3C). F, As E, but assuming mean mosquito lifespan equal to 12 days. (TIF) [file pntd.0005851.s004.tif]
